# Comprehensive analysis of NAC transcription factor family uncovers drought and salinity stress response in pearl millet (*Pennisetum glaucum*)

**DOI:** 10.1186/s12864-021-07382-y

**Published:** 2021-01-21

**Authors:** Ambika Dudhate, Harshraj Shinde, Pei Yu, Daisuke Tsugama, Shashi Kumar Gupta, Shenkui Liu, Tetsuo Takano

**Affiliations:** 1 Asian Natural Environmental Science Center (ANESC), The University of Tokyo, Nishitokyo-shi, Tokyo, 188-0002 Japan; 2 Department of Pharmaceutical Sciences, Center for Pharmaceutical Research and Innovation, College of Pharmacy, University of Kentucky, Lexington, KY USA; 3 Environmental Epigenetics and Genetics Group, Department of Horticulture, College of Agriculture, Food and Environment, University of Kentucky, Lexington, KY USA; 4 International Crops Research Institute for the Semi-Arid Tropics (ICRISAT), Hyderabad, Telangana State India; 5 State Key Laboratory of Subtropical Silviculture, Zhejiang A and F University, Lin’an, Hangzhou China

**Keywords:** Pearl millet, NAC, Transcription factor, microRNAs, Drought, Salinity

## Abstract

**Background:**

Pearl millet (*Pennisetum glaucum*) is a cereal crop that possesses the ability to withstand drought, salinity and high temperature stresses. The NAC [NAM (No Apical Meristem), ATAF1 (*Arabidopsis thaliana* Activation Factor 1), and CUC2 (Cup-shaped Cotyledon)] transcription factor family is one of the largest transcription factor families in plants. NAC family members are known to regulate plant growth and abiotic stress response. Currently, no reports are available on the functions of the NAC family in pearl millet.

**Results:**

Our genome-wide analysis found 151 NAC transcription factor genes (*PgNAC*s) in the pearl millet genome. Thirty-eight and 76 *PgNACs* were found to be segmental and dispersed duplicated respectively. Phylogenetic analysis divided these NAC transcription factors into 11 groups (A-K). Three *PgNACs* (− 073, − 29, and − 151) were found to be membrane-associated transcription factors. Seventeen other conserved motifs were found in *PgNACs*. Based on the similarity of *PgNACs* to NAC proteins in other species, the functions of *PgNACs* were predicted. In total, 88 microRNA target sites were predicted in 59 *PgNACs*. A previously performed transcriptome analysis suggests that the expression of 30 and 42 *PgNAC*s are affected by salinity stress and drought stress, respectively. The expression of 36 randomly selected *PgNAC*s were examined by quantitative reverse transcription-PCR. Many of these genes showed diverse salt- and drought-responsive expression patterns in roots and leaves. These results confirm that *PgNACs* are potentially involved in regulating abiotic stress tolerance in pearl millet.

**Conclusion:**

The pearl millet genome contains 151 NAC transcription factor genes that can be classified into 11 groups. Many of these genes are either upregulated or downregulated by either salinity or drought stress and may therefore contribute to establishing stress tolerance in pearl millet.

**Supplementary Information:**

The online version contains supplementary material available at 10.1186/s12864-021-07382-y.

## Background

Pearl millet [*Pennisetum glaucum* (L.) R. Br.] is the sixth most important staple food crop. It is a nutritionally superior crop for people living in arid and semi-arid regions of Sub-Saharan Africa and the Indian subcontinent. It can withstand harsh environmental conditions such as drought, salinity and high temperature [1]. Transcriptomic analyses has identified functional genes and pathways involved in pearl millets stress response [2–5]. The pearl millet genome has been sequenced by the International Pearl Millet Genome Sequencing Consortium [6]. The availability of pearl millet transcriptome and genome data helps to identify genes that contribute to stress tolerance in pearl millet. Among abiotic stresses, drought and salinity cause severe yield losses in major staple crops [7, 8]. Current climate prediction models forecast the deterioration of annual precipitation and an increase in salinization [9]. Breeding abiotic stress-tolerant crops such as pearl millet is therefore important to secure the food supply even under these conditions. To cope with these environmental stresses, plants activate defense responses, including the activation of sets of metabolic pathways and genes. Stress-responsive genes are classified into two types [10]: “functional” genes encoding proteins such as late embryogenesis-associated proteins, detoxification enzymes, heat shock proteins and molecular chaperones, which directly protect plants from abiotic stress, and “regulatory” genes encoding proteins such as protein kinases and transcription factors (TFs), which have roles in the perception and transduction of stress signals. TFs can interact with the promoter regions of gene and thereby alter gene expression patterns. Plant TFs are divided into different families [11]. Like many TFs, the NAC (NAM, ATAF1 and 2, and CUC2) family has versatile functions in plants [12]. The NAC domain was deduced from three previously characterized proteins, petunia NAM (no apical meristem), *Arabidopsis thaliana* ATAF1/2 (*Arabidopsis thaliana* activation factor 1/2) and CUC2 (cup-shaped cotyledon) [13, 14]. Previous studies in Arabidopsis, rice and wheat has demonstrated the involvement of NAC genes in abiotic stress responses [15–20]. A study in rice showed that, *OsNAC2* plays a positive regulatory role in drought and salt tolerance in rice through ABA-mediated pathways [21]. In wheat, NAC transcription factor, *TaNAC69* leads to enhanced tolerance to drought stress through increased expression of stress related genes [18]. Another rice NAC gene, *OsNAC05* is responsible for root diameter enlargement and drought stress tolerance [22]. Overexpression of rice stress responsive NAC gene, *SNAC1* improves drought and salt tolerance by enhancing root development and reducing transpiration rate in transgenic cotton [15]. All these study supports that NAC genes governs abiotic stress response of plants.

Genome-wide investigations of NAC transcription factor genes suggest that *Arabidopsis thaliana* has 117 NAC TFs, *Setaria italica* has 147 NAC TFs, *Oryza sativa* has 151 NAC TFs, and *Zea mays* has 152 NAC TFs [20, 23, 24]. However, NAC TF genes in pearl millet have not been analyzed thus far.

The objective of this study was to identify pearl millet NAC TFs and characterize their expression patterns. In this study, 151 NAC TF genes were identified in pearl millet (annotated as *Pennisetum glaucum* NAC genes; *PgNAC*s). We have also analyzed their genomic distribution, phylogenetic relationships, gene structure, conserved motifs, microRNA targeting and expression profiles under drought and salinity-stress conditions.

## Results

### Identification and annotations of NAC genes in pearl millet

The HMMER Search with the HMM profile identified 151 *NAC* genes among 35,757 pearl millet genes. All the identified NAC genes were named by adding the prefix “*Pg*”, for *Pennisetum glaucum*, and were numbered according to their chromosomal position, yielding *PgNAC001* to *PgNAC151*. Deduced PgNAC protein sequences exhibited a diverse range of amino acid lengths: the smallest PgNAC was 98 amino acids *(PgNAC011)* long, whereas the largest was 750 amino acids *(PgNAC134)* long (Additional File 2). The CDD search and the SMART program confirmed the presence of NAC domains in each *PgNAC*.

### Chromosomal distribution, gene structure prediction and duplication analysis of *PgNACs*

**Fig. 1:**
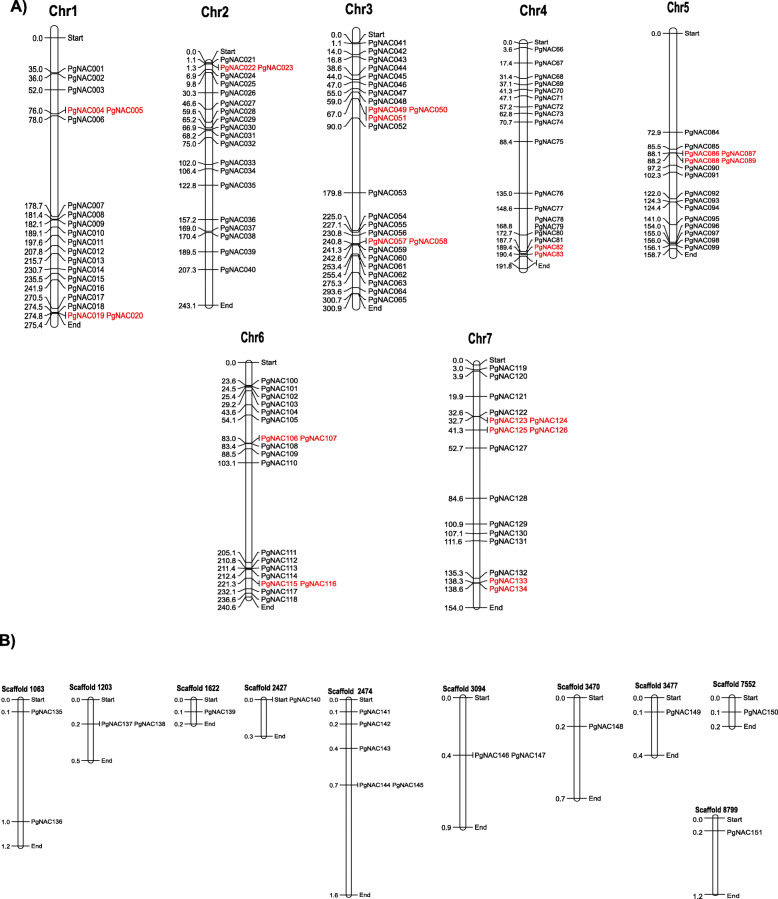
Positioning of *PgNACs* in the pearl millet genome. **a** Chromosome map showing the positions of *PgNAC001–134* on chromosomes 1–7 in pearl millet. **b** Positions of *PgNAC135*–*151* on scaffolds. Gene positions are shown in Mb. Duplicated *PgNAC*s are shown in red

**Fig. 2:**
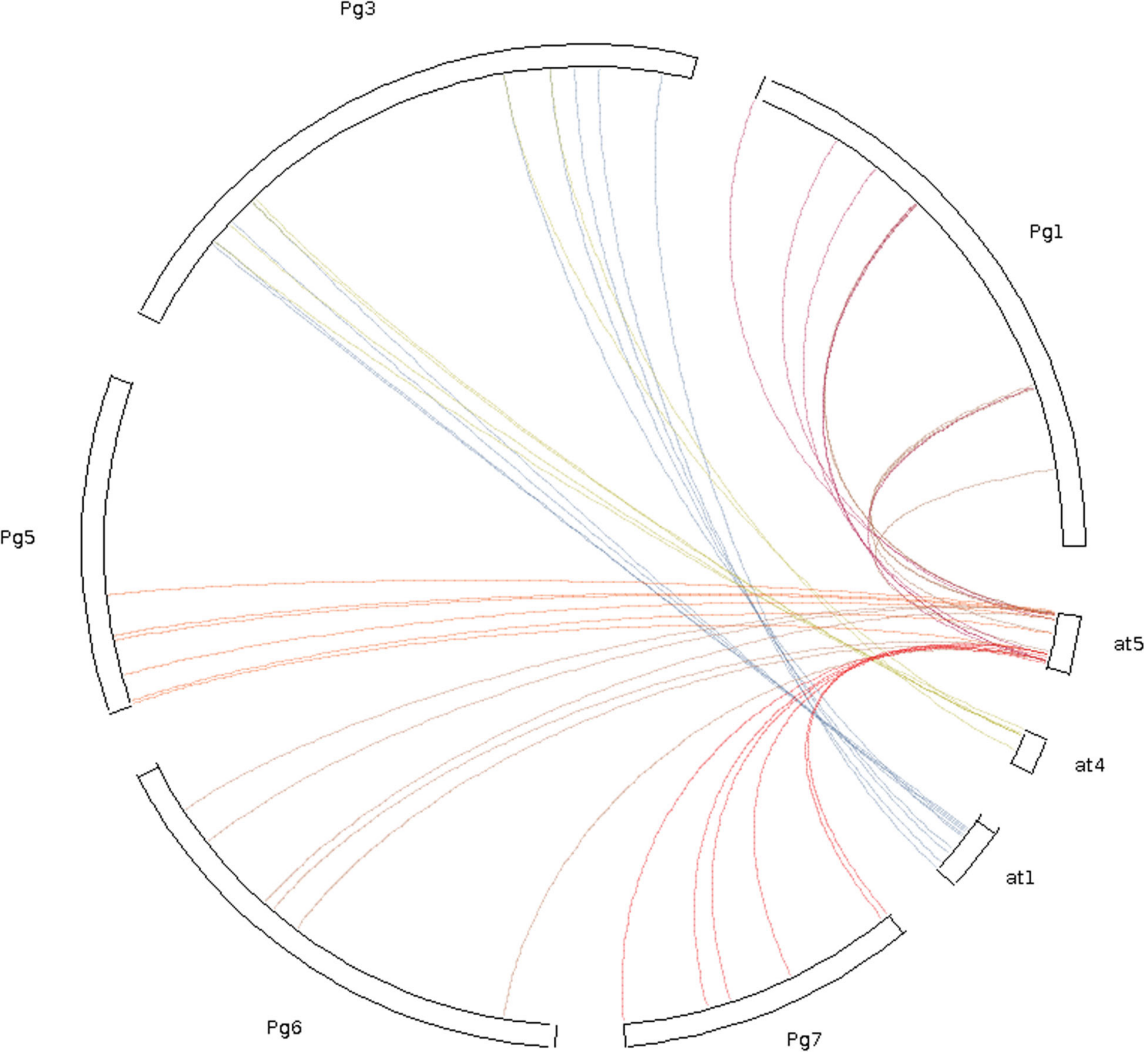
Collinearity analysis of NAC genes in pearl millet and *Arabidopsis thaliana*. The circle plot was created by MCScanX tool. Identified colinear genes were linked by the colored lines. Pg and at represent Pearl millet and *Arabidopsis thaliana* respectively

Physical mapping of *PgNAC*s on all 7 pearl millet chromosomes revealed an uneven distribution for the first 134 *PgNAC*s. Among the chromosomes, chromosome 3, which is the largest in size (300.9 Mb), had the largest number of *PgNAC*s (25; 18%). Chromosomes 1 and 6 had the second largest number (15%) of *PgNAC*s. Chromosome 5 had the smallest number (11%) of *PgNAC*s, and its size is 158.7 Mb. *PgNAC*s located on chromosomes 1 and 5 appear to congregate at one end of the arms, while chromosomes 2, 3, and 4 show clusters of *PgNAC*s at both ends. Chromosome 7 has a non-clustered distribution of *PgNAC*s. The remaining *PgNAC*s (*PgNAC135* to *PgNAC151*) mapped to 10 different scaffolds. Among these, scaffold 2474 had the largest number (5) of *PgNAC*s (*PgNAC141* to *PgNAC145*), while scaffolds 1622, 2427, 3470, 3477, 7552 and 8799 each had 1 *PgNAC* (Fig. 1). We further analyzed different types of duplication events of PgNAC genes. Among the 151 PgNAC genes 38 genes (25.17%) exhibited segmental and 76 genes (50.33%) exhibited dispersion duplication (Fig. 1 and Additional File 2). However, we didn’t find any tandem duplication in these PgNAC genes. To understand evolution and collinearity of NAC family between the species, we identified members of PgNAC family that are collinear with the model plant *Arabidopsis thaliana*. For the NAC family 32 collinear gene pairs were identified among pearl millet and *Arabidopsis thaliana* (Fig. 2).

**Fig. 3:**
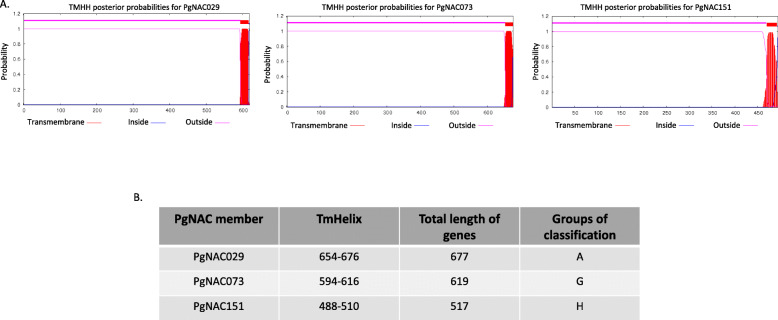
Analysis of *PgNAC* transmembrane helices. **a** Transmembrane regions in PgNAC073, PgNAC029, and PgNAC151. **b** Positions of the transmembrane helices in PgNAC073, PgNAC029, and PgNAC151, as well as their classification

**Fig. 4:**
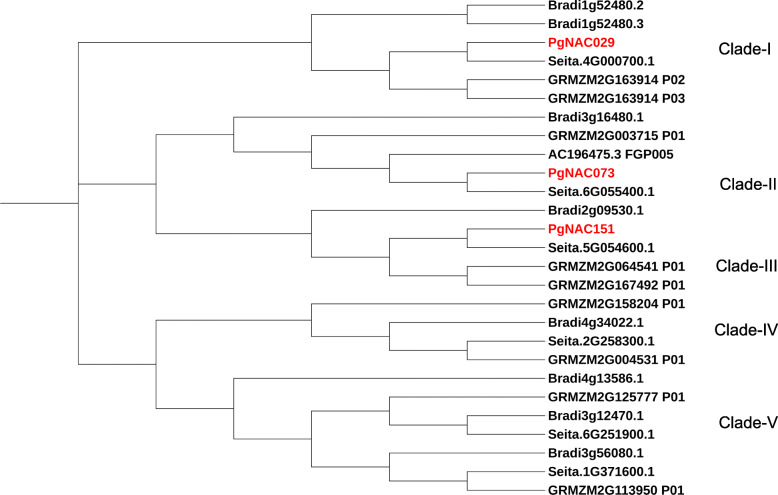
Phylogenetic relationship among membrane-associated NAC TFs from pearl millet, *Setaria italica*, *Brachypodium distachyon* and maize. Multiple sequence alignment of all putative membrane-associated NAC TFs in pearl millet (proteins with “PgNAC” in their names), *Setaria italica* (proteins with “Seita”), *Brachypodium distachyon* (proteins with “Bradi”) and maize (proteins with “GRMZM”) was conducted using ClustalW. MEGA 7.0 was used to create phylogenetic trees using the neighbor-joining method with 1000 bootstrap replicates and the p distance method

All *PgNAC*s showed great variation in size, with the smallest gene being 0.5 kb and the longest being 9 kb. The number of introns in *PgNAC*s ranged from zero to twelve. Thirty-eight *PgNAC*s consist of three exons and two introns. *PgNAC101* and *PgNAC007* have 13 and 10 exons, respectively. Twenty-nine *PgNACs* have only one exon (Additional File 3). Three *PgNACs* (*PgNAC073*, *PgNAC029*, and *PgNAC151*) were predicted to have a single transmembrane helix at the N-terminus (Fig. 3). These three *PgNACs* are more like membrane-associated NAC proteins from *Setaria italica*, *Brachypodium distachyon* and *Zea mays*. Each membrane associated *PgNAC* was placed in a different NAC protein clade (Fig. 4).

### Phylogenetic analysis of *PgNACs* and conservation of motifs

**Fig. 5:**
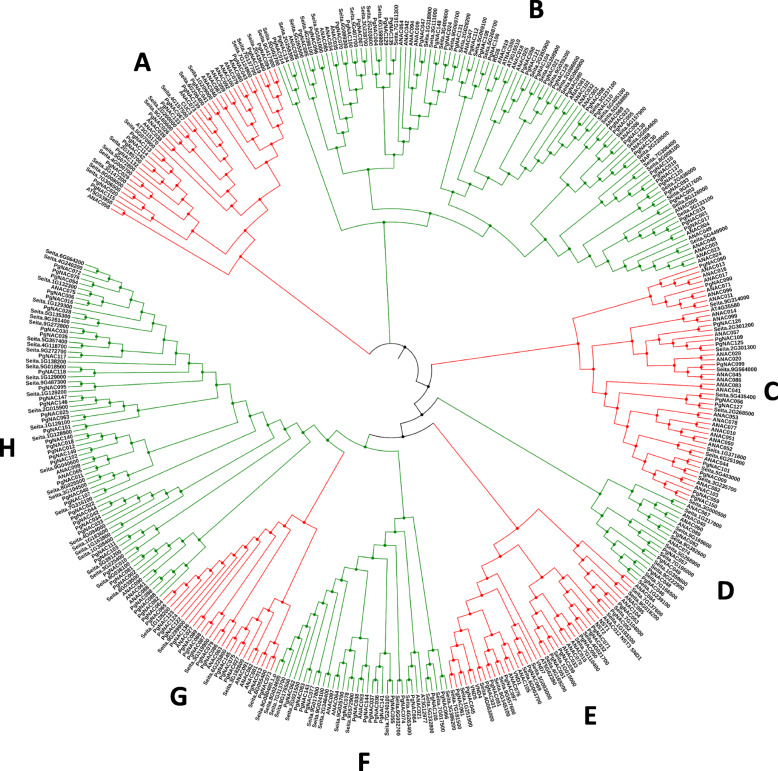
Phylogenetic analysis of pearl millet and *Setaria italica*. A phylogenetic tree was constructed using the neighbor-joining method with 1000 bootstrap replicates as described in the legend for Fig. 3. The letters A-K correspond to the 11 NAC TF subfamilies

Comprehensive phylogenetic analyses were performed by aligning the sequences of 145 *SiNACs* (*Setaria italica* NAC proteins), 126 *AtNACs* (*Arabidopsis thaliana* NAC proteins) and 151 *PgNACs*. All NACs were grouped into 8 groups (A to H). Group H was the largest group with 37 *PgNAC* members, while the smallest was group D with 5 members (Fig. 5). Most *PgNAC-*encoding genes in the same group shared similar exon-intron structures and/or duplication patterns. For instance, all the *PgNAC*-encoding members in groups D and E have 2 or 3 introns. Group B has the largest number (12) of duplicated genes.

The MEME suite found 17 motifs in *PgNACs*. *PgNAC* N-terminal regions were found to be more conserved than C-terminal regions. Generally, *PgNACs* in the same groups showed similar motif compositions (Table 1 and Additional File 4), supporting the idea that their functions are also similar.

**Table 1:** Conserved motifs present in pearl millet *PgNACs*. Conserved motifs were identified using the online tool MEME with the default parameters

No.	Motif	Sites	E-value	Width	GO annotation
1.	TCCTCCTGGATTTAGATTTCATCCTACTGATGAWGAACTTRTTRNTYATT	130	5.4e-1077	50	BP: Multicellular organism development
2.	AGAMCTAATAGAGCTACTGVWDCTGGATATTGGAAGGCTACTGGAAMKGA	74	1.7e-852	50	CC: chloroplastMF: RNA binding
3.	TTGGAATGAAGAAGACTCTTGTTTTTTATAGAGGAAGAGCTCCTAAGGGA	77	7.1e-802	50	MF: oxygen binding
4.	TGTTATTGCTGAAGTTGATATTTATAAGTTTGATCCTTGGGATCTTCCTG	149	3.9e-805	50	
5.	AAGACTGATTGGATTATGCATGAATATAGACTTGAAGATGCTGATGATGC	128	6.6e-773	50	
6.	AGGAATGGTATTTTTTTTCTCCTAGAGATAGAAAGTATCCTAATGGAGCT	87	1.6e-640	50	
7.	CTGCTGCTCCTGCTCCTGCTCCTATTGTTATTGCTCAAGCTGCTGCTCCT	145	1.2e-566	50	
8.	CTGCTGCTGGAGGAGGAGAAGGATCTTCTTCTGAAGCTGCTGCTGCTGCT	145	5.8e-452	50	CC: mitochondrionCC: chloroplast thylakoid membraneCC: anchored to membrane
9.	TAAGGAAGATTGGGTTCTTTGTAGAGTTTTTTATAAGTCTAGAGCTACTA	134	3.8e-395	50	
10.	TACTCTTACTCATGATTCTGTTATGCCTTCTACTGCTGCTCAAGTTTCTG	125	2.6e-338	50	MF: RNA bindingCC: chloroplast thylakoid membraneMF: transcription factor activityBP: protein transportMF: ATP binding
11.	GCTCCTCCTCCTCCTCCTCCT	143	7.5e-212	21	
12.	TTCCTAAGGTTGAACCTCAAGCTGATGATGGAGGAAATTCTCTTGCTGCT	125	1.3e-259	50	
13.	CTTCTTGCTGATACTACTTCTGGAGCTTTTCAATATTCTTCTCTTCTTT	125	1.6e-172	49	CC: plasma membrane
14.	TTAAGCTTGCTGGAGAAGCTCTTCCTGCTGCTGCTGGATCT	121	3.6e-121	41	
15.	AGAAATTTCTTCTTCTTCTGATTATCTTAAGCTTCCTCCTGAACCTGCTG	78	6.3e-104	50	
16.	TCTTGCTCCTAAGGCTGCTGATGCTGGA	145	1.4e-093	28	CC: chloroplast
17.	GCTGATCCTTCTTCTGCTCCTGTTAAGGCTAAGAGACAAC	53	2.2e-067	40	

### *PgNACs* GO annotation

**Fig. 6:**
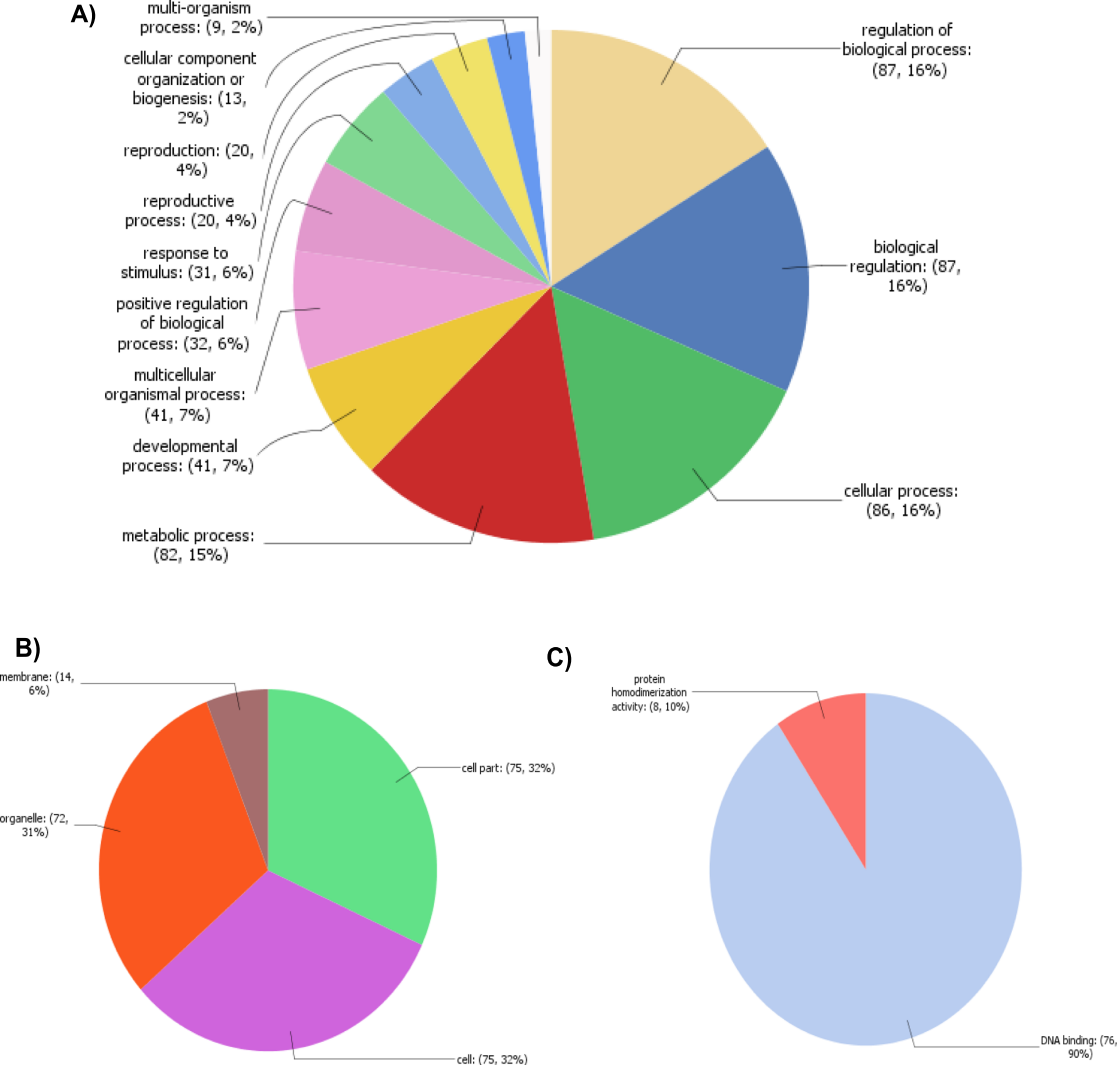
Gene Ontology (GO) terms associated with PgNACs. **a** GO terms for the category “Biological process”. **b** GO terms for the category “Molecular functions”. **c** GO terms for the category “Cellular components”

GO term analysis suggested that the majority of *PgNACs* are involved in DNA binding (90%). Eight *PgNACs* (*PgNAC009, PgNAC021, PgNAC085, PgNAC125, PgNAC029, PgNAC112, PgNAC118,* and *PgNAC137*) were associated with the GO term “homodimerization”. Approximately 86.25% of *PgNACs* were associated with the GO term “Nucleus”. Nine *PgNACs* were associated with the GO term “external and internal stimuli”, and 29 were associated with “response to stress” (Fig. 6).

### miRNAs target sites in *PgNACs*

The results of analyzing miRNAs targeting *PgNACs* found a total of 88 miRNA target sites in the 59 *PgNAC*s. Among them, *PgNAC023* had the most (six) miRNA target sites. *PgNAC023* is targeted by miRNA162, miRNA167h, miRNA394a and miRNA394b. *PgNAC092* had four miRNA target sites, and is targeted by miR165a, miR165b, miR166b and miR166p. Among miRNAs, miR529 had the most (17) target sites in 14 PgNAC genes. In a previous study, miR529 was shown to regulate resistance to oxidative stress by targeting transcription factor genes in rice [25]. (Additional file: 5). Our findings suggest that expression of *PgNACs* is regulated by multiple miRNAs.

### Transcriptomic expression of *PgNACs* during drought and salinity stress

**Fig. 7:**
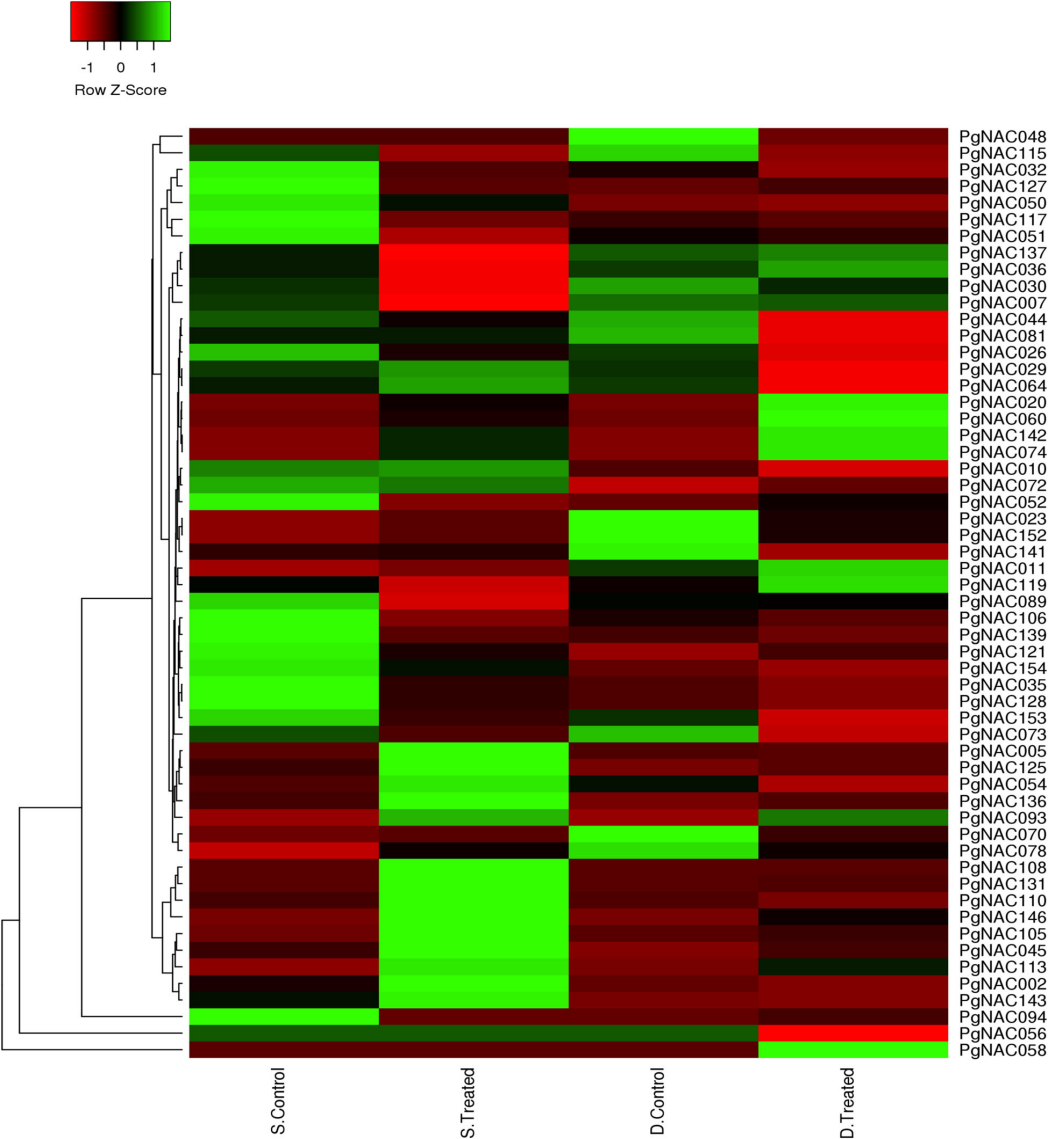
*PgNAC* expression levels based on a previous transcriptome analysis. The colors in the heat map reflect *PgNAC* expression levels. The samples used included roots from non-stressed plants (S. Control); roots from salinity-stressed roots (S. Treated), roots from non-stressed roots (D. Control), and roots from drought-stressed plants (D. Treated)

*PgNAC* expression was examined using previously published transcriptome data. Seventy-two *PgNAC*s were expressed under these drought-stressed and salinity-stressed conditions. *PgNAC108*, *PgNAC131*, *PgNAC110*, *PgNAC146*, *PgNAC105*, *PgNAC045*, *PgNAC113*, *PgNAC002*, *PgNAC143*, *PgNAC005*, *PgNAC125*, *PgNAC054*, and *PgNAC136* were strongly expressed under salinity-stressed conditions. Whereas *PgNAC137*, *PgNAC036*, *PgNAC007*, *PgNAC020*, *PgNAC060*, *PgNAC142*, *PgNAC074*, and *PgNAC011* were strongly expressed under drought-stressed conditions. *PgNAC093*, *PgNAC142*, *PgNAC074*, *PgNAC020*, and *PgNAC060* were expressed under both salinity and drought conditions (Fig. 7).

### Expression profiling by qRT-PCR

**Fig. 8:**
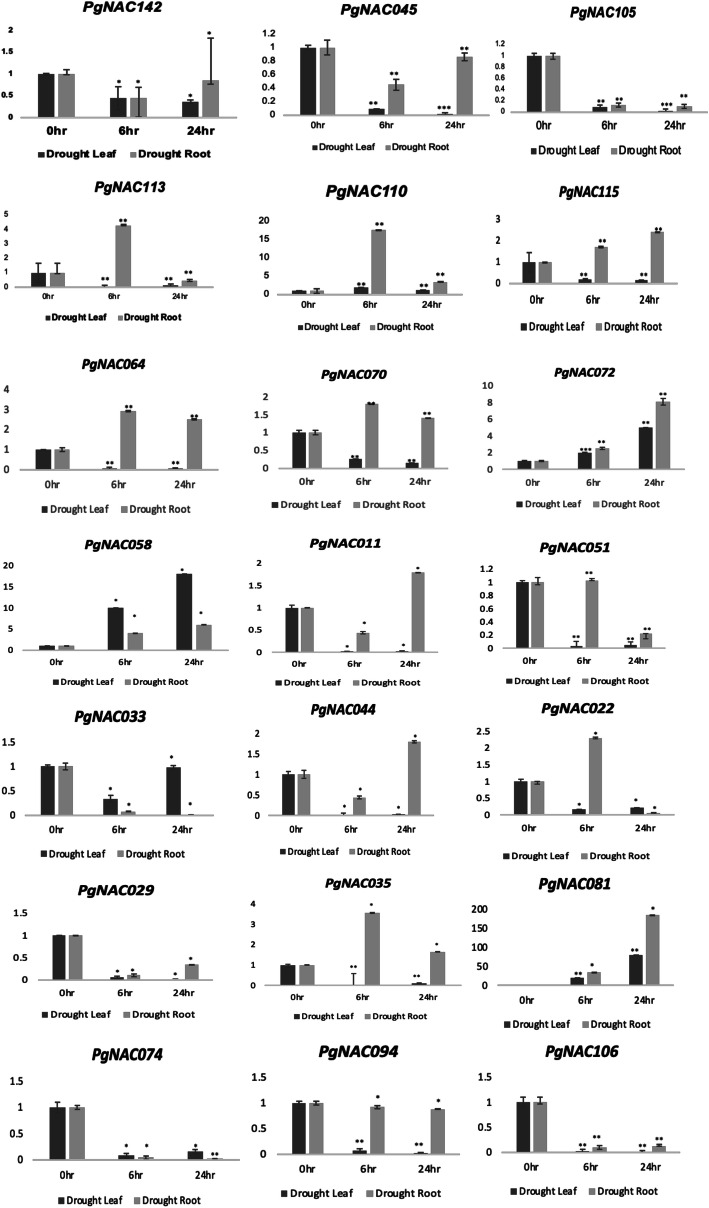
Expression of selected *PgNAC*s under drought-stressed conditions. Pearl millet plants were exposed to drought stress for 0, 6, and 24 h, and their roots and leaves were sampled. The relative mRNA abundance of 21 selected *PgNAC*s in these samples was determined by quantitative reverse transcription-PCR, with *PgActin* as an internal control. Data are presented as the means ± standard deviations (SD) of three biological replicates. Asterisk indicate significant differences. * *P* < 0.05, **P < 0.01, ***P < 0.001, students t-test

**Fig. 9:**
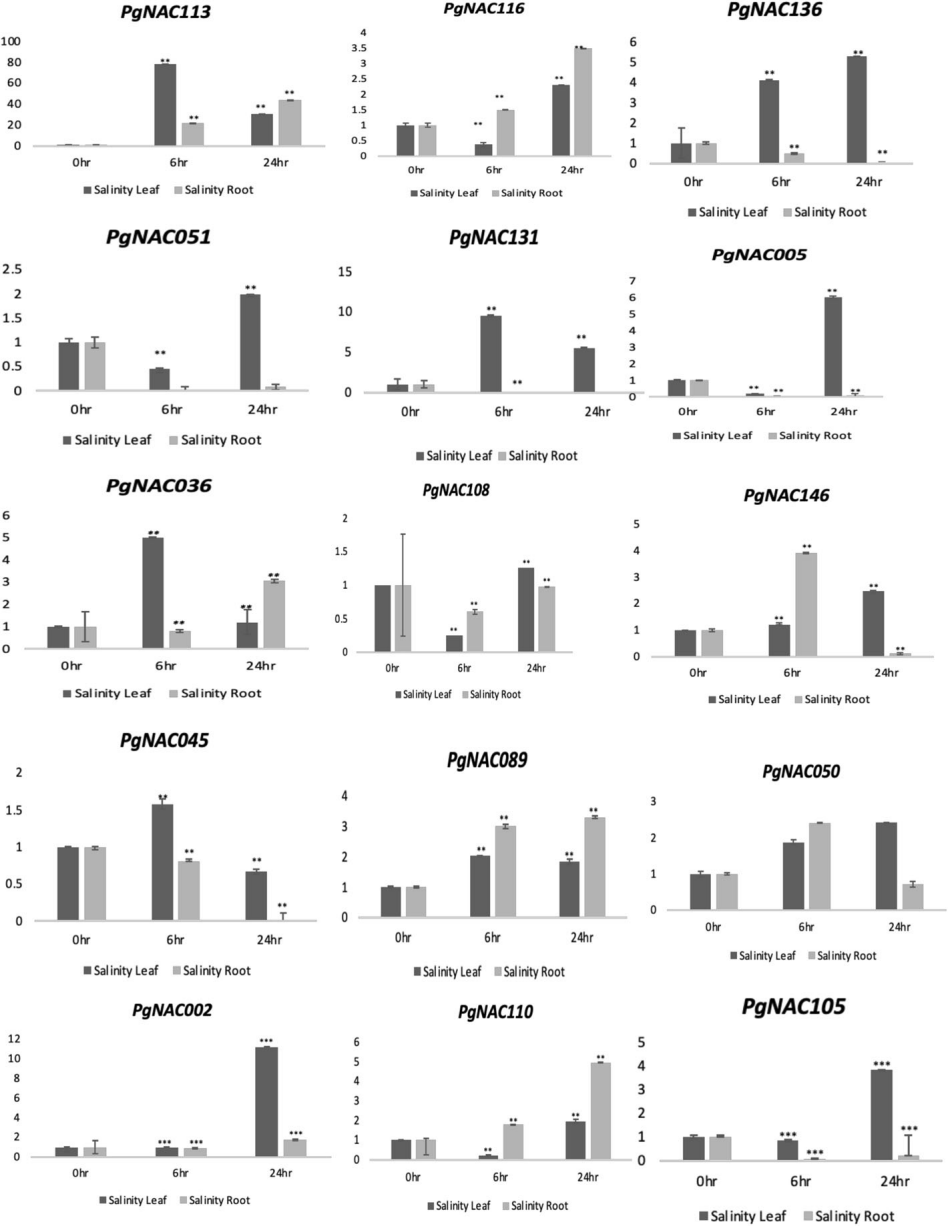
Expression analysis of selected *PgNAC*s for salinity. Pearl millet plants were exposed to salinity stress for 0, 6, and 24 h, and their roots and leaves were sampled. The relative mRNA abundance of 16 selected *PgNAC*s was determined in these samples using quantitative reverse transcription-PCR, with *PgActin* as an internal control. Data are presented as the means ± standard deviations (SD) of three biological replicates. Asterisk indicate significant differences. * *P* < 0.05, **P < 0.01, ***P < 0.001, students t-test

Quantitative RT-PCR was performed to confirm the expression of randomly selected *PgNAC*s under drought and salinity stress. For drought, *PgNAC142*, *PgNAC045*, *PgNAC105*, *PgNAC113*, *PgNAC110*, *PgNAC072*, *PgNAC044*, *PgNAC011*, *PgNAC022*, *PgNAC051*, *PgNAC029*, *PgNAC094*, *PgNAC106*, *PgNAC074*, *PgNAC033*, *PgNAC035*, and *PgNAC081* were studied. The expression of *PgNAC081* was 170 times higher in roots under drought-stressed condition than control condition. Under drought-stressed conditions, most *PgNAC*s were more strongly expressed in roots than in leaves (Fig. 8). *PgNAC029*, *PgNAC106*, and *PgNAC074* were downregulated in both leaf and root tissues. For salinity, *PgNAC051*, *PgNAC005*, *PgNAC036*, *PgNAC116*, *PgNAC146*, *PgNAC108 PgNAC131*, *PgNAC093*, *PgNAC089*, *PgNAC050*, *PgNAC136*, *PgNAC002*, *PgNAC113*, *PgNAC018*, *PgNAC105*, *PgNAC045* and *PgNAC110* were selected. Most of these *PgNAC*s were upregulated in both root and leaf tissues. *PgNAC113* was strongly induced in roots by salinity stress (with 76-fold change) than control condition. (Fig. 9).

## Discussion

The chromosomal distribution of *PgNAC*s was uneven and clustered. This pattern is similar to the distribution of NAC TF genes in *Setaria italica*, *Oryza sativa*, *Solanum tuberosum*, *Brachypodium distachyon* and other species [24, 26–28]. Structurally diversified multigene family are known to develop during evolution to cope with changing environmental conditions [29]. The numbers of introns in *PgNAC*s ranges from 0 to 12, which is comparable to NAC TF genes in rice (0–16) [26]. However, banana and cassava NAC TFs contain 0–5 and 0–9 introns, respectively [30, 31]. During gene duplication, the loss of introns occurs faster than the gain of introns [26]. The 29 *PgNAC*s that have no introns may represent the original genes. In plants, segmental and dispersion duplication contributes to evolution of novel functions such as adaptation to stress [32]. In this study, 75% PgNAC genes exhibited either segmental or dispersion duplication. In miRNA-*PgNACs* association studies 88 miRNAs binding sites were found in 59 *PgNACs*. This association studies confirms that miRNAs are master regulators of *PgNACs*. Most of these pearl millet miRNAs targeting *PgNACs* are salinity and drought stress responsive. Previous studies have proved that miRNAs and NAC TFs together are key players of stress responses [33, 34]. However, further studies are necessary to elucidate the roles of these miRNAs and their association with pearl millet NACs.

Using qRT-PCR, we identified 36 *PgNAC*s that respond to either drought or salinity stress. Some of the drought and salinity stress responsive PgNACs shows homology with previously reported stress responsive NAC genes [15, 28]. In our analysis, many *PgNAC*s were upregulated in roots rather than in leaves. This difference may be because roots are the primary organ exposed to stress [2]. Similar results were obtained for NAC TF genes in *Brachypodium distachyon* when their expression levels were assessed after plants were exposed to cadmium stress [35]. In common bean (*Phaseolus vulgaris*), 11 NAC TF genes were upregulated by drought stress, while 8 were downregulated [36]. JUNGBRUNNEN 1 (JUB1), a NAC factor, acts as an important regulator of drought tolerance in *Solanum lycopersicum* [37]. Another *Arabidopsis* NAC TF, *ANAC092*, regulates senescence, seed germination, and tolerance to salt stress [38]. Several other NAC TFs, such as *OsNAC6* [16, 39, 40] in rice; *ANAC019*, *ANAC056* and *ANAC072* [41] in *Arabidopsis*; and *SNAC1* in cotton [15], are involved in establishing stress tolerance in plants. Similar to these NAC TFs, *PgNACs* are likely to regulate drought and salinity tolerance in pearl millet. In a previous study, one of the stress-responsive pearl millet NAC transcription factor gene, *PgNAC21*, was overexpressed in *Arabidopsis*, and improved its salinity stress tolerance [42]. In the future, we plan to perform functional characterization of other PgNAC genes. The stress responsive PgNAC genes identified in our study could be utilized as promising candidates for molecular breeding to generate new stress tolerant plant genotypes.

## Conclusion

In our study, a comprehensive analysis including gene structure, phylogenetic analysis, chromosomal location, miRNA binding and expression analysis under abiotic stresses of the NAC gene family in pearl millet was first performed. We identified 151 NAC genes in the pearl millet genome, these genes provide preliminary information for the future functional characterization studies. Furthermore, expression analysis of PgNAC genes in root and leaf tissues and under salinity and drought stresses implied that *PgNACs* participate in the stress responses of the pearl millet. Our systematic analysis of the NAC TF genes in pearl millet lays foundation for the follow-up study of the functional characteristics of PgNAC genes and the cultivation of stress tolerant pearl millet varieties. In future, we will study functions of some of the key PgNACs by overexpressing them in pearl millet or *Arabidopsis thaliana*.

## Methods

### Sequence retrieval, database searches and domain searches

To identify the *PgNAC*s, 35,756 coding gene sequences of pearl millet were retrieved from the GIGA database (http://gigadb.org/dataset/100192) and converted to all 6 standard reading frames with the standalone JEMBOSS software [43, 44]. Nucleotide sequences for the 1016 NAC TFs from *Arabidopsis thaliana*, *Oryza sativa*, *Setaria italica*, *Sorghum bicolor* and *Zea mays* were retrieved from the Plant Transcription Factor Database v4.0 (http://planttfdb.cbi.pku.edu.cn/family.php?fam=NAC) [11] and used as subjects to perform BLAST searches against the pearl millet coding sequences. The hidden Markov model (HMM) profile of NAC and NAM domains was extracted from the pFAM database [45–47], and NAC and NAM HMM profiles were used to search the pearl millet genome sequences for specific domains using HMMER [48]. Hits with an expected value less than 1.0 were selected, and redundancy was minimized using the decrease redundancy tool (https://web.expasy.org/decrease_redundancy). The presence of NAC domains in the filtered sequences was confirmed by SMART (http://smart.embl-heidelberg.de) and pFAM (http://pfam.xfam.org/). For the transmembrane motifs, the sequences were searched using the TMHMM server ver 2.0 (http://www.cbs.dtu.dk/services/TMHMM/).

### Chromosome positioning, gene structure and gene duplication analysis for PgNACs

The BLASTN program in the BLAST+ suite [49] was performed using *PgNAC* sequences as queries and the pearl millet genome sequence (Taxid: 4543) as the database to determine the positions of *PgNAC*s in the pearl millet genome. The positions of *PgNAC*s in the genome were displayed using MapChart [50]. Gene structure display server program (GSDS, http://gsds.cbi.pku.edu.cn/index.php) was utilized to show exon/intron structure of each PgNAC gene by comparing the coding sequences with their corresponding genomic sequences [51]. NAC sequences of *Arabidopsis thaliana* were extracted from TAIR database [52]. MCScanX was employed to analyze various types of duplications including segmental, tandem, proximal and dispersed with default parameters. Members of NAC gene family with the segmental duplication and collinear analysis were retrieved from the above datasets for further analysis [53].

### Phylogenetic analysis and conserved motif identification

For phylogenetic analysis, the sequences of all NAC transcription factors were imported into MEGA7, and multiple sequence alignment was performed using CLUSTALW with the default’s parameters. A file with the extension “.MEG” was used to construct an unrooted phylogenetic tree based on the neighbor-joining method using bootstrap analysis with 1000 replicates [54]. The MEGA7 output tree was edited using the Interactive Tree of Life online tool (iTOL; https://itol.embl.de/). Conserved motifs in the NAC transcription factor sequences were identified using multiple EM for motif elicitation (MEME suite). The input parameters for motif identification were as follows: number of repetitions, any; maximum number of motifs, 20; and width of motif, 50. The discovered motifs were annotated using GOMo gene ontology [55].

### Prediction of microRNAs target sites in *PgNACs*

In pearl millet, microRNAs (miRNAs) have been reported to be involved in stress responses [3, 56]. MiRNAs are small (20–24 nucleotides) non-coding RNAs derived from single-stranded precursors. MiRNAs negatively regulate target genes by pairing to the corresponding gene and facilitating its cleavage [57]. The psRNATarget tool was used to identify the target sites of pearl millet miRNAs in *PgNAC*s (http://plantgrn.noble.org/psRNATarget/) [58]. To avoid false-positives, only gene sequences with a 3.0 points cut-off threshold were considered miRNAs target genes.

### NAC TF GO annotation and expression analysis using transcriptome data

Gene Ontology (GO) terms were assigned to *PgNACs* using the Blast2GO program based on their similarities to *Oryza sativa* proteins (National Center for Biotechnology Information (NCBI) taxid: 4530) [59].

Previously available RNA sequencing data for drought and salinity conditions were primarily used to examine the expression of the *PgNAC*s. These data were retrieved from the NCBI Sequence Read Archive under accession numbers SRR6327851 to SRR6327856; SRR6327857 to SRR6327862 for drought-stressed samples and SRP128956 for salinity-stressed samples [2, 4].

### Quantitative reverse transcription-PCR (qRT-PCR)

Drought- (ICMB843) and salinity-tolerant (ICMB01222) pearl millet genotypes were obtained from the International Crops Research Institute for the Semi-Arid Tropics (ICRISAT), India. Eexperiments were conducted under controlled greenhouse conditions. Approximately, 20 seeds from each genotype were sown in equal volumes of soil and vermiculite in perforated terracotta pots. Each growth experiment was performed in triplicate. Drought stress was imposed on 21-day-old plants by incubating the plants in 20% (w/v) PEG for 6 and 24 h, while control plants were maintained in normal water. For salinity stress, plants were incubated in 250 mM NaCl solution for 6 and 24 h. Total RNA was isolated from root and leaf tissues with Trizol. For cDNA synthesis, 1 μg of total RNA was reverse transcribed with oligo (dT) primers and PrimeScript Reverse Transcriptase. To examine *PgNAC* genes expression, real-time PCR was performed using a step-one real-time instrument and the TB Green I kit (Roche, Basel, Switzerland). Reactions were performed in triplicate and each reaction mixture contained 100 ng of cDNA, 1 μL of each primer (10 μM/μL) and 10 μL of TB green master mix in a final volume of 20 μL. The primers sequences used for qRT-PCR are provided in (Additional File 1). The PCR conditions were as follows: initial denaturation at 95 °C for 30 s, 40 cycles of 95 °C for 5 s and 60 °C for 30 s, and melt-curve analysis. The *PgActin* gene was used as a reference [60]. The qPCR assays were performed with three replicates. Relative fold differences for each sample in each experiment were calculated using the ΔΔCt method [61].

## Supplementary Information

**Additional file 1.** List of primers used in quantitative real time-PCR expression analysis of PgNAC genes.

**Additional file 2.** Detail catalogue of NAC in pearl millet.

**Additional file 3 **Gene structure of *PgNACs*. Image was created by submitting the sequences to the gene structure display 2.0 online tool. Red boxes denote the exon/coding region, black lines are intron and blue boxes defines.

**Additional file 4 **Location of 17 Motifs on the *PgNACs*. The location of motifs were derived by the multiple alignment of the motifs by online tool MAST. Each of the following 152 sequences has an *E*-value less than 10. The motif matches shown here have a position *p*-value less than 0.0001. Colored boxes represent the respective motif.

**Additional file 5.** Analyzing pearl millet miRNAs targeting PgNACs.

## Data Availability

All data generated or analyzed during this study are included in this published article [and its supplementary information files].
