# Supplementary material for: Comprehensive analysis of NAC transcription factor family uncovers drought and salinity stress response in pearl millet (Pennisetum glaucum)
Source: BMC Genomics. 2021 Jan 21;22:70. doi: 10.1186/s12864-021-07382-y (PMC7818933; doi:10.1186/s12864-021-07382-y)
Supplement: Supplementary file 1 — Additional file 1. List of primers used in quantitative real time-PCR expression analysis of PgNAC genes. [file 12864_2021_7382_MOESM1_ESM.doc]

**Additional File 1.** List of primers used in quantitative real time-PCR expression analysis of PgNAC genes.

| **Condition** | **Gene** | **Forward primer** | **Reverse Primer** |
| --- | --- | --- | --- |
| **Drought** | PgNAC142  PgNAC045  PgNAC105  PgNAC113  PgNAC110  PgNAC115  PgNAC064  PgNAC070  PgNAC072  PgNAC058  PgNAC044  PgNAC011  PgNAC022  PgNAC051  PgNAC029  PgNAC094  PgNAC106  PgNAC074  PgNAC035  PgNAC081 | GGGGCGAGAAGACCAACT  ACAAGTTCGTCGCGTCTCA  TTCAGCAAGTGCCAGGTGT  GGCGCTCGTCTTCTACTCCG  TTCAGGTTCCATCCCACG  GACGGGCGAGTTCATCCA  CTTTACAGAGGCTCTAGGAG  ATGACCAACCAAACCAGGAGA  TCACTTTGGCTCCTACTGCG  CCAGACATCGTCCCAACA  GACAAGAACGACTGCCAAGC  ATCGGGACCAGGAAGACCAT  TAATCAGGCGACGGAAAGA  GCAGTGGCTGTATTGTTG  GTGCAGGGTGTTCAAGAAGTG  TTTCCACGAATCCGACAT  TGCTCTTCGTGGAGACGC  CAGCAACAGCAGCTTATTGGA  GCCATCTACACCTCGTCCCG  AATGGGTGGGATAGCAGTAGCA | GCACCATGTCCAGCGTGT  TTCCACAGGTCGTTATCCC  CGGTGACGGGTTTCATTCC  CAGCACCCACTCGTCCAACT  CATCATCTTCGCCTTGCTT  CCCAGGTTCCCTCCTTGTT  TCATCCGTTTCAGACTTG  CAGGTGCCAGACGATGAGC  CAGCGGGTCCGTGTTGTAC  AGCCACCACTTTCTACCAC  CGCCGTGAAGTCCAAACC  TCCAGAGCCTTGTACTCGTTGA  AACCACCAGCAGCGAAAT  CCTCTTCCTTTGGGATTT  GCTGAGGATGGAGGAGACG  TAAAGAAGAACCAGGAGCAG  ACCTGGGCTGGGAGTAGGAC  GCAGATTGGTGGTCGTCGTA  GTACTCGTGCATGATCCAGTCG  ACGGAGGGCGCACAAAGA |
| **Salinity** | PgNAC051  PgNAC005  PgNAC036  PgNAC116  PgNAC146  PgNAC045  PgNAC131  PgNAC105  PgNAC093  PgNAC089  PgNAC050  PgNAC136  PgNAC002  PgNAC110  PgNAC108  PgNAC113 | GCAGTGGCTGTATTGTTG  AGGAGCACAAGAACGAGC  TCCGTGGTCAGAAGAAGCAA  GCAAGGTGTACCGCAACCC  ACGGGTAAGGATAAGGAGA  ACAAGTTCGTCGCGTCTCA  GGGGTCAAGACGGAGTGGA  TTCAGCAAGTGCCAGGTGT  TTTCCACGAATCCGACAT  AGCAAGCGGCTGCCAACT  AGCAGAACCACCACGGCGAGAC  CGACCGCGTGGTACTTCT  GACAAGGCCATCGTTTCG  TTCAGGTTCCATCCCACG  GCGGTGAGAAGAGCAACTGG  GGCGCTCGTCTTCTACTCCG | CCTCTTCCTTTGGGATTT  TGCCCAGAAATAGGGAGA  TGAGGTGTCCGCGTATGG  GCCGCCTCTTCCTCATCAA  GTTTGAGCTTGATGTGGG  TTCCACAGGTCGTTATCCC  TTGTGGTACAGGCGGCAGA  CGGTGACGGGTTTCATTCC  TAAAGAAGAACCAGGAGCAG  CATGATTTCATCGAGGTCCAAA  AAGGCGGCGAGGTAGGCATC  TTCACCTCTTTCCTGCCTGT  TGCAGTGAGGAGGCGGTAC  CATCATCTTCGCCTTGCTT  TTGTAGGAGGCTTTGGTGGG  CAGCACCCACTCGTCCAACT |
