# Supplementary figures and images for: Comprehensive analysis of NAC transcription factor family uncovers drought and salinity stress response in pearl millet (Pennisetum glaucum)
Source: BMC Genomics. 2021 Jan 21;22:70. doi: 10.1186/s12864-021-07382-y (PMC7818933; doi:10.1186/s12864-021-07382-y)

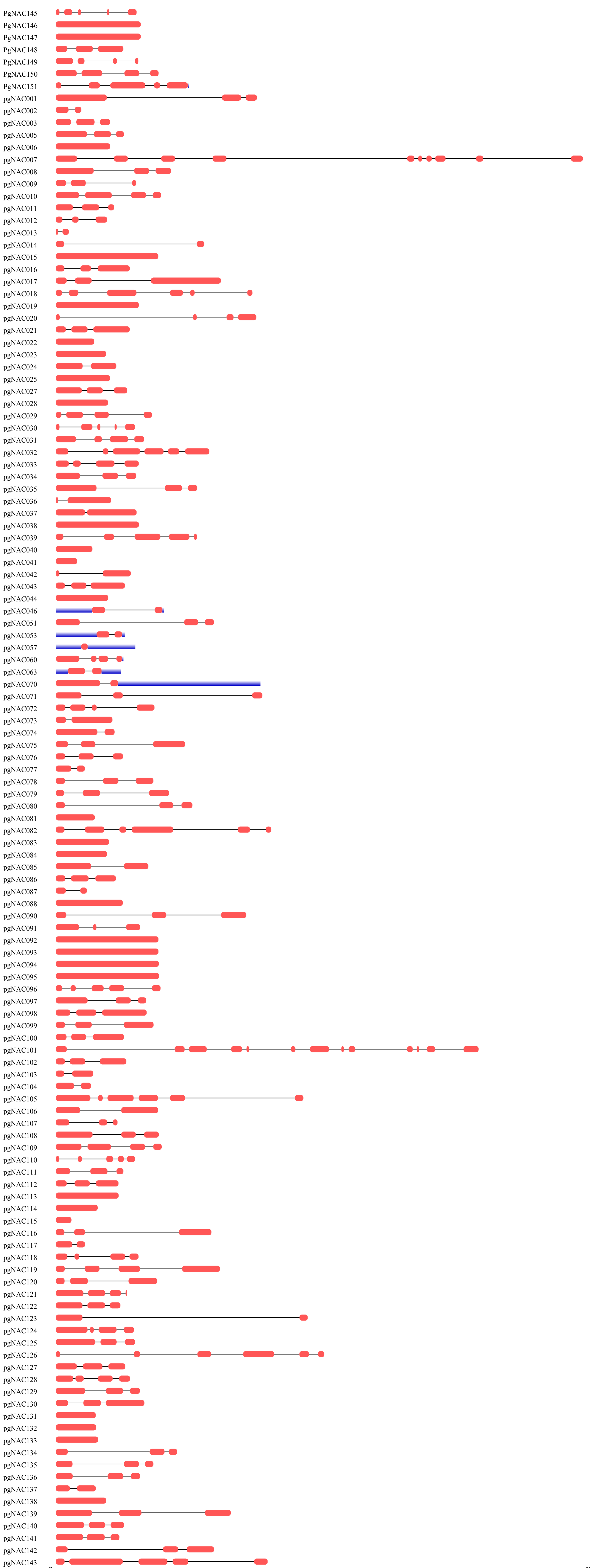

Supplement: Supplementary file 3 — Additional file 3 Gene structure of PgNACs. Image was created by submitting the sequences to the gene structure display 2.0 online tool. Red boxes denote the exon/coding region, black lines are intron and blue boxes defines. [file 12864_2021_7382_MOESM3_ESM.pdf]
