# Supplementary material for: Comprehensive analysis of NAC transcription factor family uncovers drought and salinity stress response in pearl millet (Pennisetum glaucum)
Source: BMC Genomics. 2021 Jan 21;22:70. doi: 10.1186/s12864-021-07382-y (PMC7818933; doi:10.1186/s12864-021-07382-y)
Supplement: Supplementary file 4 — Additional file 4 Location of 17 Motifs on the PgNACs. The location of motifs were derived by the multiple alignment of the motifs by online tool MAST. Each of the following 152 sequences has an E-value less than 10. The motif matches shown here have a position p-value less than 0.0001. Colored boxes represent the respective motif. [file 12864_2021_7382_MOESM4_ESM.pdf]

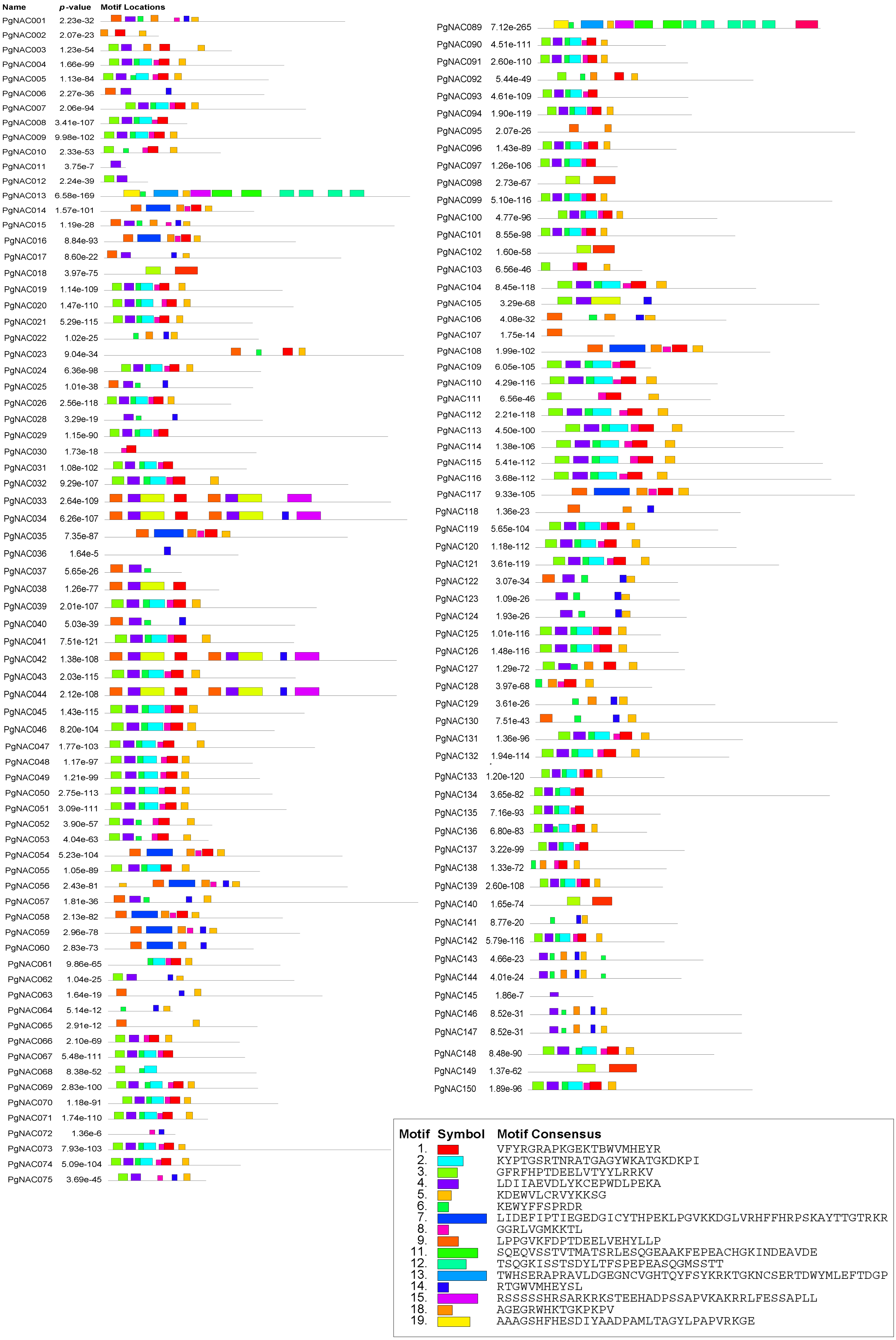
Name p-value Motif Locations
PgNAC001 2.23e-32
PgNAC002 2.07e-23
PgNAC003 1.23e-54
PgNAC004 1.66e-99
PgNAC005 1.13e-84
PgNAC006 2.27e-36
PgNAC007 2.06e-94
PgNAC008 3.41e-107
PgNAC009 9.98e-102
PgNAC010 2.33e-53
PgNAC011 3.75e-7
PgNAC012 2.24e-39
PgNAC013 6.58e-169
PgNAC014 1.57e-101
PgNAC015 1.19e-28
PgNAC016 8.84e-93
PgNAC017 8.60e-22
PgNAC018 3.97e-75
PgNAC019 1.14e-109
PgNAC020 1.47e-110
PgNAC021 5.29e-115
PgNAC022 1.02e-25
PgNAC023 9.04e-34
PgNAC024 6.36e-98
PgNAC025 1.01e-38
PgNAC026 2.56e-118
PgNAC028 3.29e-19
PgNAC029 1.15e-90
PgNAC030 1.73e-18
PgNAC031 1.08e-102
PgNAC032 9.29e-107
PgNAC033 2.64e-109
PgNAC034 6.26e-107
PgNAC035 7.35e-87
PgNAC036 1.64e-5
PgNAC037 5.65e-26
PgNAC038 1.26e-77
PgNAC039 2.01e-107
PgNAC040 5.03e-39
PgNAC041 7.51e-121
PgNAC042 1.38e-108
PgNAC043 2.03e-115
PgNAC044 2.12e-108
PgNAC045 1.43e-115
PgNAC046 8.20e-104
PgNAC047 1.77e-103
PgNAC048 1.17e-97
PgNAC049 1.21e-99
PgNAC050 2.75e-113
PgNAC051 3.09e-111
PgNAC052 3.90e-57
PgNAC053 4.04e-63
PgNAC054 5.23e-104
PgNAC055 1.05e-89
PgNAC056 2.43e-81
PgNAC057 1.81e-36
PgNAC058 2.13e-82
PgNAC059 2.96e-78
PgNAC060 2.83e-73
PgNAC061 9.86e-65
PgNAC062 1.04e-25
PgNAC063 1.64e-19
PgNAC064 5.14e-12
PgNAC065 2.91e-12
PgNAC066 2.10e-69
PgNAC067 5.48e-111
PgNAC068 8.38e-52
PgNAC069 2.83e-100
PgNAC070 1.18e-91
PgNAC071 1.74e-110
PgNAC072 1.36e-6
PgNAC073 7.93e-103
PgNAC074 5.09e-104
PgNAC075 3.69e-45
PgNAC089 7.12e-265
PgNAC090 4.51e-111
PgNAC091 2.60e-110
PgNAC092 5.44e-49
PgNAC093 4.61e-109
PgNAC094 1.90e-119
PgNAC095 2.07e-26
PgNAC096 1.43e-89
PgNAC097 1.26e-106
PgNAC098 2.73e-67
PgNAC099 5.10e-116
PgNAC100 4.77e-96
PgNAC101 8.55e-98
PgNAC102 1.60e-58
PgNAC103 6.56e-46
PgNAC104 8.45e-118
PgNAC105 3.29e-68
PgNAC106 4.08e-32
PgNAC107 1.75e-14
PgNAC108 1.99e-102
PgNAC109 6.05e-105
PgNAC110 4.29e-116
PgNAC111 6.56e-46
PgNAC112 2.21e-118
PgNAC113 4.50e-100
PgNAC114 1.38e-106
PgNAC115 5.41e-112
PgNAC116 3.68e-112
PgNAC117 9.33e-105
PgNAC118 1.36e-23
PgNAC119 5.65e-104
PgNAC120 1.18e-112
PgNAC121 3.61e-119
PgNAC122 3.07e-34
PgNAC123 1.09e-26
PgNAC124 1.93e-26
PgNAC125 1.01e-116
PgNAC126 1.48e-116
PgNAC127 1.29e-72
PgNAC128 3.97e-68
PgNAC129 3.61e-26
PgNAC130 7.51e-43
PgNAC131 1.36e-96
PgNAC132 1.94e-114
PgNAC133 1.20e-120
PgNAC134 3.65e-82
PgNAC135 7.16e-93
PgNAC136 6.80e-83
PgNAC137 3.22e-99
PgNAC138 1.33e-72
PgNAC139 2.60e-108
PgNAC140 1.65e-74
PgNAC141 8.77e-20
PgNAC142 5.79e-116
PgNAC143 4.66e-23
PgNAC144 4.01e-24
PgNAC145 1.86e-7
PgNAC146 8.52e-31
PgNAC147 8.52e-31
PgNAC148 8.48e-90
PgNAC149 1.37e-62
PgNAC150 1.89e-96
Motif Symbol Motif Consensus
1. VFYRGRAPKGEKTBWVMHEYR
2. KYPTGSRTNRATGAGYWKATGKDKPI
3. GFRFHPTDEELVTYYLRRKV
4. LDIIAEVDLYKCEPWDLPEKA
5. KDEWVLCRVYKKSG
6. KEWYFFSPRDR
7. LIDEFIPTIEGEDGICYTHPEKLPGVKKDGLVRHFFHRPSKAYTTGTRKR
8. GGRLVGMKKTL
9. LPPGVKFDPTDEELVEHYLLP
11. SQEQVSSTVTMATSRLESQGEAAKFEPEACHGKINDEAVDE
12. TSQGKISSTSDYLTFSPEPEASQGMSSTT
13. TWHSERAPRAVLDGEGNCVGHTQYFSYKRKTGKNCSERTDWYMLEFTDGP
14. RTGWVMHEYSL
15. RSSSSSHRSARKRKSTEEHADPSSAPVKAKRRLFESSAPLL
18. AGEGRWHKTGKPKPV
19. AAAGSHFHESDIYAADPAMLTAGYLPAPVRKGE
